# Supplementary material for: Telehealth versus face-to-face visits: A comprehensive outpatient perspective-based cohort study of patients with kidney disease
Source: PLoS One. 2022 Mar 11;17(3):e0265073. doi: 10.1371/journal.pone.0265073 (PMC8916620; doi:10.1371/journal.pone.0265073)
Supplement: S1 File — S1 Table: Number of available patient surveys per diagnosis group. Distribution of diagnostic categories and survey responses; S2 Table: Diagnoses Grouping of patients seen in Outpatient nephrology March–July 2020. Overall diagnoses by number of patients seen in the division of Nephrology March through July 2020; S3 Table: Outpatient Nephrology Survey. Patient- facing survey tool; S4 Table: How the frequency of top box score of 5 varied by month for telenephrology survey responses. Variability of top box score: 5 only, reported by month; S1 Fig: Press Ganey Survey—Medical Practice Survey Response Rate for October 2019 –September 2020, Nephrology Division, Mayo Clinic, Rochester MN. Graphical form of the variability of the top box scores (4 or 5) by month; S5 Table: Press Ganey Survey—Medical Practice Survey Response Rate for October 2019 –September 2020, Nephrology Division, Mayo Clinic, Rochester MN. Nephrology provider numbers in the division of Nephrology and the survey response rates; S2 Fig: Survey respondents distribution by visit type and diagnoses groups (Face-to-face and telenephrology). Respondents by diagnostic groups; S6 Table: Survey Respondents by primary diagnosis group. Distribution of respondents by face to face and telemedicine. (PDF) [file pone.0265073.s001.pdf]

Supporting Information

Supplemental Table 1: Number of available patient surveys per diagnosis group

Group name	Group ID number	Number of surveys responses
AKI	1	6
Bone mineral disease	2	1
BP ailments	3	56
CKD I-III	4	143
CKD IV -V	5	66
Cystic kidney disease	6	18
Diabetes	7	8
Fluid and electrolyte imbalances	8	13
Hematuria	9	13
Malignancy	10	6
Nephrolithiasis	11	34
Other	12	53
Renal Parenchymal disease	13	36
Urological ailments	14	9
Total		462

Supporting Information

Supplemental Table 2: Diagnoses Grouping of patients seen in Outpatient nephrology March – July 2020

Primary visit diagnoses	Count of Primary Diagnosis ICD 10
**AKI**	**151**
Acute kidney failure with tubular necrosis (HCC)	13
Acute kidney failure, unspecified (HCC)	133
Other acute kidney failure (HCC)	5
**Bone mineral disease**	**38**
Age-related osteoporosis without current pathological fracture	4
Disorder of parathyroid gland, unspecified (HCC)	2
Disorder of phosphorus metabolism, unspecified	1
Hyperparathyroidism, unspecified (HCC)	9
Hypoparathyroidism, unspecified (HCC)	1
Other disorders of calcium metabolism	7
Other disorders of phosphorus metabolism	5
Other specified disorders of bone density and structure, unspecified site	4
Primary hyperparathyroidism (HCC)	5
**BP ailments**	**773**
Acute on chronic right heart failure (HCC)	1
Autonomic dysreflexia	2
Biventricular heart failure (HCC)	1
Chronic combined systolic (congestive) and diastolic (congestive) heart failure (HCC)	1
Conn's syndrome (HCC)	1
Disorder of adrenal gland, unspecified (HCC)	4
Disorder of the autonomic nervous system, unspecified	10
Elevated blood-pressure reading, without diagnosis of hypertension	31
Essential (primary) hypertension	631
Heart disease, unspecified	1
Heart failure, unspecified (HCC)	1
Hyperaldosteronism, unspecified (HCC)	5
Hypertension secondary to endocrine disorders	8
Hypertensive emergency	4
Hypertensive encephalopathy	2
Hypertensive heart disease with heart failure (HCC)	5
Hypertensive heart disease without heart failure	10
Hypertensive urgency	6
Hypotension, unspecified	7
Left ventricular failure, unspecified (HCC)	2
Occlusion and stenosis of bilateral carotid arteries	1
Occlusion and stenosis of right carotid artery	1
Orthostatic hypotension	8
Other hypertrophic cardiomyopathy (HCC)	1
Other primary hyperaldosteronism (HCC)	3
Other secondary hypertension	9
Other specified disorders of adrenal gland (HCC)	1
Other specified endocrine disorders	1
Pre-existing essential hypertension complicating pregnancy, second trimester	2
Pre-existing essential hypertension complicating pregnancy, unspecified trimester	1

Supporting Information

Right heart failure, unspecified (HCC)	1
Secondary hypertension, unspecified	9
Supervision of other high risk pregnancies, unspecified trimester	1
Twin pregnancy, monochorionic/diamniotic, second trimester	1
**CKD I-III**	**1088**
Anemia, unspecified	9
Chronic kidney disease, stage 1	3
Chronic kidney disease, stage 2 (mild)	51
Chronic kidney disease, stage 3 (moderate) (HCC)	628
Chronic kidney disease, unspecified	16
Hypertensive chronic kidney disease with stage 1 through stage 4 chronic kidney disease, or unspecified chronic kidney disease	348
Hypertensive heart and chronic kidney disease with heart failure and stage 1 through stage 4 chronic kidney disease, or unspecified chronic kidney disease (HCC)	18
Hypertensive heart and chronic kidney disease without heart failure, with stage 1 through stage 4 chronic kidney disease, or unspecified chronic kidney disease	7
Iron deficiency anemia secondary to blood loss (chronic)	2
Iron deficiency anemia, unspecified	1
Nutritional anemia, unspecified	2
Secondary hyperparathyroidism of renal origin (HCC)	1
Secondary hyperparathyroidism, not elsewhere classified (HCC)	1
Unspecified kidney failure	1
**CKD IV -V**	**757**
Chronic kidney disease, stage 4 (severe) (HCC)	518
Chronic kidney disease, stage 5 (HCC)	216
Hypertensive chronic kidney disease with stage 5 chronic kidney disease or end stage renal disease (HCC)	15
Hypertensive heart and chronic kidney disease with heart failure and with stage 5 chronic kidney disease, or end stage renal disease (HCC)	3
Hypertensive heart and chronic kidney disease without heart failure, with stage 5 chronic kidney disease, or end stage renal disease (HCC)	2
Secondary hyperparathyroidism of renal origin (HCC)	3
**Cystic kidney disease**	**207**
Congenital multiple renal cysts	1
Cyst of kidney, acquired	19
Cystic disease of liver	13
Cystic kidney disease, unspecified	5
Family history of polycystic kidney	1
Medullary cystic kidney	9
Other cystic kidney diseases	1
Other polycystic kidney, infantile type (HCC)	2
Polycystic kidney, adult type	132
Polycystic kidney, unspecified	24
**Diabetes**	**89**
Diabetes mellitus due to underlying condition with diabetic nephropathy (HCC)	6
Diabetes mellitus due to underlying condition with hyperglycemia (HCC)	1
Hyperglycemia, unspecified	5
Impaired glucose tolerance (oral)	1
Metabolic syndrome	1

Supporting Information

Other abnormal glucose	1
Other specified diabetes mellitus with diabetic nephropathy (HCC)	1
Other specified diabetes mellitus with other diabetic kidney complication (HCC)	1
Prediabetes	1
Pre-existing type 1 diabetes mellitus, in pregnancy, second trimester	1
Type 1 diabetes mellitus with diabetic chronic kidney disease (HCC)	1
Type 1 diabetes mellitus with diabetic nephropathy (HCC)	4
Type 1 diabetes mellitus without complications (HCC)	8
Type 2 diabetes mellitus with diabetic chronic kidney disease (HCC)	9
Type 2 diabetes mellitus with diabetic nephropathy (HCC)	21
Type 2 diabetes mellitus with diabetic neuropathy, unspecified (HCC)	1
Type 2 diabetes mellitus with hyperglycemia (HCC)	1
Type 2 diabetes mellitus with moderate nonproliferative diabetic retinopathy without macular edema, bilateral (HCC)	1
Type 2 diabetes mellitus with other diabetic kidney complication (HCC)	2
Type 2 diabetes mellitus with other skin ulcer (HCC)	1
Type 2 diabetes mellitus with other specified complication (HCC)	3
Type 2 diabetes mellitus with severe nonproliferative diabetic retinopathy with macular edema, bilateral (HCC)	1
Type 2 diabetes mellitus without complications (HCC)	17
**Fluid and electrolyte imbalances**	**187**
Acidosis	1
Alkalosis	1
Amyloidosis, unspecified (HCC)	2
Bartter's syndrome (HCC)	1
Dehydration	2
Diarrhea, unspecified	1
Disorders of magnesium metabolism, unspecified	2
Edema, unspecified	11
Gestational edema with proteinuria, first trimester	1
Hypercalcemia	14
Hyperkalemia	31
Hyperosmolality and hypernatremia	2
Hypocalcemia	2
Hypokalemia	24
Hypomagnesemia	17
Hypo-osmolality and hyponatremia	13
Hypovolemia	1
Localized edema	13
Other disorders of electrolyte and fluid balance, not elsewhere classified	1
Other fluid overload	6
Other specified abnormal findings of blood chemistry	40
Vomiting, unspecified	1
**Hematuria**	**156**
Abnormal uterine and vaginal bleeding, unspecified	1
Asymptomatic microscopic hematuria	1
Benign essential microscopic hematuria	2
Gross hematuria	6
Hematuria, unspecified	50
Other microscopic hematuria	2

Supporting Information

Recurrent and persistent hematuria with other morphologic changes	94
**Malignancy**	**85**
Acute lymphoblastic leukemia not having achieved remission (HCC)	1
Acute myelomonocytic leukemia, not having achieved remission (HCC)	1
Benign neoplasm of left kidney	1
Benign neoplasm of right kidney	3
Benign neoplasm of thymus	1
Bone marrow transplant status (HCC)	1
Chronic myeloproliferative disease (HCC)	1
Encounter for antineoplastic immunotherapy	2
Follicular lymphoma grade ii, intra-abdominal lymph nodes (HCC)	1
Light chain (AL) amyloidosis (HCC)	13
Lymphangioleiomyomatosis (HCC)	1
Malignant neoplasm of bladder, unspecified (HCC)	4
Malignant neoplasm of colon, unspecified (HCC)	1
Malignant neoplasm of connective and soft tissue, unspecified (HCC)	1
Malignant neoplasm of descended left testis (HCC)	1
Malignant neoplasm of endometrium (HCC)	1
Malignant neoplasm of left kidney, except renal pelvis (HCC)	3
Malignant neoplasm of lower third of esophagus (HCC)	1
Malignant neoplasm of nasopharynx, unspecified (HCC)	1
Malignant neoplasm of oropharynx, unspecified (HCC)	1
Malignant neoplasm of pancreatic duct (HCC)	1
Malignant neoplasm of posterior wall of bladder (HCC)	1
Malignant neoplasm of prostate (HCC)	3
Malignant neoplasm of rectum (HCC)	1
Malignant neoplasm of right kidney, except renal pelvis (HCC)	3
Malignant neoplasm of right renal pelvis (HCC)	1
Malignant neoplasm of unspecified part of left bronchus or lung (HCC)	1
Malignant neoplasm of unspecified site of unspecified female breast (HCC)	1
Malignant neoplasm of upper lobe, left bronchus or lung (HCC)	1
Mesothelioma of pleura (HCC)	1
Monoclonal gammopathy	13
Multiple myeloma not having achieved remission (HCC)	1
Neoplasm of uncertain behavior of lymphoid, hematopoietic and related tissue, unspecified (HCC)	1
Organ-limited amyloidosis (HCC)	1
Other specified diseases of blood and blood-forming organs	2
Personal history of malignant neoplasm of thyroid	2
Personal history of other malignant neoplasm of kidney	4
Secondary systemic amyloidosis (HCC)	6
Waldenstrom macroglobulinemia (HCC)	1
**Nephrolithiasis**	**449**
Calculus in bladder	2
Calculus of kidney	344
Calculus of kidney with calculus of ureter	41
Calculus of ureter	2
Cystinuria (HCC)	3
Disorder resulting from impaired renal tubular function, unspecified	1
Hypercalciuria	5

Supporting Information

Hyperoxaluria	10
Hyperuricemia without signs of inflammatory arthritis and tophaceous disease	2
Hypocitraturia	1
Personal history of urinary calculi	6
Primary hyperoxaluria (HCC)	12
Unspecified renal colic	1
Urinary calculus, unspecified	19
**Other**	**598**
Abdominal aortic aneurysm, without rupture (HCC)	3
Abdominal distension (gaseous)	1
Abnormal electrocardiogram (ECG) (EKG)	1
Abnormal findings on diagnostic imaging of other abdominal regions, including retroperitoneum	1
Abnormal immunological findings in specimens from other organs, systems and tissues	1
Abnormal results of liver function studies	1
Abnormal weight loss	3
Acne, unspecified	1
Acquired absence of stomach (part of)	1
Acute bronchiolitis, unspecified	1
Acute pyelonephritis	4
Acute sinusitis, unspecified	1
Aneurysm of renal artery (HCC)	1
Aortic aneurysm of unspecified site, without rupture (HCC)	1
Arterial fibromuscular dysplasia (HCC)	9
Arteritis, unspecified (HCC)	4
Atherosclerosis of aorta (HCC)	1
Atherosclerosis of native arteries of extremities with intermittent claudication, bilateral legs (HCC)	1
Atherosclerosis of native arteries of extremities with intermittent claudication, left leg (HCC)	1
Atherosclerosis of renal artery (HCC)	29
Atherosclerotic heart disease of native coronary artery with other forms of angina pectoris (HCC)	1
Atherosclerotic heart disease of native coronary artery with unstable angina pectoris (HCC)	1
Atherosclerotic heart disease of native coronary artery without angina pectoris	4
Bacteremia	1
Benign paroxysmal vertigo, bilateral	1
Bradycardia, unspecified	2
Burn of unspecified degree of abdominal wall, initial encounter	1
Cardiac arrhythmia, unspecified	2
Cardiac murmur, unspecified	1
Cardiomyopathy, unspecified (HCC)	3
Cellulitis, unspecified	1
Cerebral infarction, unspecified (HCC)	4
Chest pain, unspecified	2
Chronic diastolic (congestive) heart failure (HCC)	1
Chronic obstructive pulmonary disease with (acute) lower respiratory infection (HCC)	2
Complications of stem cell transplant (HCC)	1

Supporting Information

Congenital insufficiency of aortic valve (HCC)	1
Constipation, unspecified	1
Cough	2
Cushing's syndrome, unspecified (HCC)	1
Decreased white blood cell count, unspecified	1
Deficiency of other specified B group vitamins	1
Disease of spinal cord, unspecified (HCC)	1
Disorder of the skin and subcutaneous tissue, unspecified	2
Disorientation, unspecified	1
Dissection of renal artery (HCC)	1
Disseminated intravascular coagulation (defibrination syndrome) (HCC)	1
Diverticulosis of large intestine without perforation or abscess without bleeding	1
Dizziness and giddiness	2
Dorsalgia, unspecified	2
Dyspnea, unspecified	1
Encounter for antenatal screening for chromosomal anomalies	1
Encounter for examination for normal comparison and control in clinical research program	2
Encounter for follow-up examination after completed treatment for conditions other than malignant neoplasm	1
Encounter for general adult medical examination without abnormal findings	8
Encounter for other preprocedural examination	13
Encounter for screening, unspecified	1
Essential tremor	1
Fatty (change of) liver, not elsewhere classified	2
Female infertility, unspecified	1
Fever, unspecified	2
Finding of unspecified substance, not normally found in blood	2
Gastro-esophageal reflux disease without esophagitis	4
Generalized anxiety disorder	1
Generalized hyperhidrosis	1
Glycosuria	1
Gout, unspecified	6
Heart transplant status (HCC)	1
Heartburn	1
Hemochromatosis, unspecified	1
Hepatorenal syndrome (HCC)	1
Hyperlipidemia, unspecified	16
Hypersensitivity angiitis (HCC)	1
Hypothyroidism, unspecified	5
Immunodeficiency, unspecified (HCC)	2
Infection following a procedure, unspecified, initial encounter	1
Inflammatory polyps of colon without complications (HCC)	1
Interstitial pulmonary disease, unspecified (HCC)	3
Intra-abdominal and pelvic swelling, mass and lump, unspecified site	3
Irritable bowel syndrome with diarrhea	2
Ischemia and infarction of kidney (HCC)	2
Left lower quadrant abdominal swelling, mass and lump	1
Left upper quadrant pain	1
Liver disease, unspecified	1

Supporting Information

Liver transplant status (HCC)	6
Long term (current) use of anticoagulants	3
Low back pain	7
Lung transplant status (HCC)	1
Lymphedema, not elsewhere classified	2
Malabsorption due to intolerance, not elsewhere classified (HCC)	1
Mixed irritable bowel syndrome	1
Morbid (severe) obesity due to excess calories (HCC)	5
Multiple endocrine neoplasia (MEN) type I (HCC)	1
Nausea	1
Nicotine dependence, cigarettes, uncomplicated	1
Noninfective gastroenteritis and colitis, unspecified	1
Non-pressure chronic ulcer of other part of right lower leg limited to breakdown of skin (HCC)	1
Non-pressure chronic ulcer of unspecified part of left lower leg with unspecified severity (HCC)	1
Nonrheumatic aortic (valve) insufficiency	1
Nonrheumatic aortic (valve) stenosis	4
Nonrheumatic aortic valve disorder, unspecified	2
Nonrheumatic mitral (valve) insufficiency	1
Nontraumatic intracerebral hemorrhage, unspecified (HCC)	1
Obesity, unspecified	4
Obstructive sleep apnea (adult) (pediatric)	1
Other atherosclerosis of native arteries of extremities, bilateral legs (HCC)	1
Other chest pain	3
Other chronic pain	1
Other chronic pancreatitis (HCC)	2
Other diseases of capillaries	1
Other diseases of mediastinum, not elsewhere classified	2
Other disorders resulting from impaired renal tubular function	9
Other fatigue	5
Other forms of dyspnea	4
Other general symptoms and signs	4
Other long term (current) drug therapy	4
Other phakomatoses, not elsewhere classified (HCC)	1
Other specified abnormal immunological findings in serum	12
Other specified cardiac arrhythmias	1
Other specified diseases of liver	7
Other specified disorders of arteries and arterioles (HCC)	43
Other specified disorders of nose and nasal sinuses	1
Other specified erythematous conditions	1
Other specified hearing loss, bilateral	1
Other specified personal risk factors, not elsewhere classified	3
Other specified postprocedural states	1
Other specified soft tissue disorders	2
Other specified symptoms and signs involving the circulatory and respiratory systems	4
Other vascular disorders of intestine (HCC)	1
Other visual disturbances	1
Overweight	1

Supporting Information

Pain in left hip	1
Pain in right hip	1
Pain in right knee	1
Pain in unspecified joint	2
Palpitations	1
Paroxysmal atrial fibrillation (HCC)	2
Periodic paralysis	1
Personal history of other diseases of the circulatory system	14
Personal history of other diseases of the musculoskeletal system and connective tissue	1
Personal history of other specified conditions	1
Personal history of transient ischemic attack (TIA), and cerebral infarction without residual deficits	1
Pigmentary retinal dystrophy	1
Pneumonia, unspecified organism	1
Polymyalgia rheumatica (HCC)	1
Polyneuropathy, unspecified	7
Postsurgical malabsorption, not elsewhere classified (HCC)	2
Presence of coronary angioplasty implant and graft	1
Primary adrenocortical insufficiency (HCC)	2
Pulmonary hypertension, unspecified (HCC)	2
Pure hypercholesterolemia, unspecified	1
Pyuria	4
Radiculopathy, site unspecified	1
Rash and other nonspecific skin eruption	1
Raynaud's syndrome without gangrene	1
Renal agenesis, unilateral	1
Renal sclerosis, unspecified	57
Renovascular hypertension	37
Right upper quadrant pain	1
Schizoaffective disorder, unspecified (HCC)	1
Secondary polycythemia	5
Secondary pulmonary arterial hypertension (HCC)	1
Shortness of breath	2
Sicca syndrome with other organ involvement (HCC)	1
Sleep apnea, unspecified	1
Sleep disorder, unspecified	1
Snoring	2
Spinal stenosis, lumbar region with neurogenic claudication	2
Spontaneous ecchymoses	1
ST elevation (STEMI) myocardial infarction involving left anterior descending coronary artery (HCC)	1
Stem cells transplant status (HCC)	1
Stricture of artery (HCC)	2
Supervision of high risk pregnancy, unspecified, unspecified trimester	2
Syncope and collapse	4
Syndrome of inappropriate secretion of antidiuretic hormone (HCC)	3
Tachycardia, unspecified	1
Thoracic aortic aneurysm, ruptured (HCC)	1
Thoracic aortic aneurysm, without rupture (HCC)	1

Supporting Information

Thrombotic microangiopathy (HCC)	5
Transient cerebral ischemic attack, unspecified	1
Tremor, unspecified	1
Tuberous sclerosis (HCC)	4
Underweight	1
Unilateral primary osteoarthritis, right knee	1
Unspecified abdominal pain	12
Unspecified abnormal findings in urine	1
Unspecified atrial fibrillation (HCC)	3
Unspecified atrioventricular block	1
Unspecified convulsions (HCC)	1
Unspecified dementia without behavioral disturbance (HCC)	1
Unspecified diastolic (congestive) heart failure (HCC)	2
Unspecified open wound, left lower leg, initial encounter	1
Urinary tract infection, site not specified	15
Varicose veins of bilateral lower extremities with other complications	1
Weakness	2
**Renal Parenchymal disease**	**439**
Acute nephritic syndrome with diffuse crescentic glomerulonephritis	1
Acute nephritic syndrome with other morphologic changes	2
Acute nephritic syndrome with unspecified morphologic changes	3
Allergic purpura (HCC)	1
Alport syndrome (HCC)	3
Arthropathic psoriasis, unspecified (HCC)	1
Chronic nephritic syndrome with diffuse crescentic glomerulonephritis	2
Chronic nephritic syndrome with diffuse mesangiocapillary glomerulonephritis	1
Chronic nephritic syndrome with focal and segmental glomerular lesions	4
Chronic nephritic syndrome with other morphologic changes	2
Chronic nephritic syndrome with unspecified morphologic changes	1
Chronic tubulo-interstitial nephritis, unspecified	3
Cr(e)st syndrome (HCC)	1
Cryoglobulinemia (HCC)	2
Defects In The Complement System (HCC)	2
Discoid lupus erythematosus	4
Fabry (-anderson) disease (HCC)	2
Glomerular disease in systemic lupus erythematosus (HCC)	55
Hemolytic-uremic syndrome (HCC)	4
Inflammatory polyarthropathy (HCC)	1
Isolated proteinuria with diffuse endocapillary proliferative glomerulonephritis	1
Isolated proteinuria with focal and segmental glomerular lesions	1
Lupus anticoagulant syndrome (HCC)	1
Microscopic polyangiitis (HCC)	3
Nephrotic syndrome with dense deposit disease	6
Nephrotic syndrome with diffuse membranous glomerulonephritis	6
Nephrotic syndrome with diffuse mesangiocapillary glomerulonephritis	3
Nephrotic syndrome with other morphologic changes	1
Nephrotic syndrome with unspecified morphologic changes	12
Other chronic tubulo-interstitial nephritis	3
Other nonthrombocytopenic purpura (HCC)	1
Proteinuria, unspecified	78

Supporting Information

Psoriasis, unspecified	1
Recurrent and persistent hematuria with diffuse membranous glomerulonephritis	1
Recurrent and persistent hematuria with unspecified morphologic changes	2
Rheumatoid arthritis, unspecified (HCC)	1
Sarcoidosis of lung with sarcoidosis of lymph nodes (HCC)	1
Sarcoidosis of other sites	2
Sarcoidosis, unspecified	2
Sicca syndrome with tubulo-interstitial nephropathy (HCC)	2
Sicca syndrome, unspecified (HCC)	2
Systemic lupus erythematosus, unspecified (HCC)	25
Tubulo-interstitial nephritis, not specified as acute or chronic	12
Unspecified nephritic syndrome with dense deposit disease	3
Unspecified nephritic syndrome with diffuse membranous glomerulonephritis	68
Unspecified nephritic syndrome with diffuse mesangiocapillary glomerulonephritis	31
Unspecified nephritic syndrome with focal and segmental glomerular lesions	4
Unspecified nephritic syndrome with minor glomerular abnormality	37
Unspecified nephritic syndrome with other morphologic changes	21
Unspecified nephritic syndrome with unspecified morphologic changes	12
Wegener's granulomatosis without renal involvement (HCC)	1
**Urological ailments**	**93**
Acquired absence of kidney	13
Atrophy of kidney (terminal)	4
Benign prostatic hyperplasia with lower urinary tract symptoms	1
Benign prostatic hyperplasia without lower urinary tract symptoms	2
Bladder disorder, unspecified	1
Congenital malformation of kidney, unspecified	1
Congenital renal failure	1
Disorder of kidney and ureter, unspecified	28
Dysuria	1
Elevated prostate specific antigen (PSA)	3
Family history of disorders of kidney and ureter	1
Flaccid neuropathic bladder, not elsewhere classified	1
Frequency of micturition	2
Lobulated, fused and horseshoe kidney	1
Neuromuscular dysfunction of bladder, unspecified	2
Obstructive and reflux uropathy, unspecified	4
Other artificial openings of urinary tract status (HCC)	4
Other difficulties with micturition	1
Other polyuria	1
Other specified disorders of kidney and ureter	4
Personal history of other diseases of urinary system	2
Retention of urine, unspecified	1
Spermatocele of epididymis, unspecified	1
Unspecified hydronephrosis	6
Unspecified urinary incontinence	3
Urethral syndrome, unspecified	1
Urgency of urination	1
Vesicoureteral-reflux with reflux nephropathy without hydroureter, bilateral	1
Vesicoureteral-reflux with reflux nephropathy without hydroureter, unspecified	1
**Grand Total**	**5110**

Supporting Information

Supplemental table 3: Outpatient Nephrology Survey

MAYO CLINIC

MEDICAL PRACTICE TELEMEDICINE SURVEY

We thank you in advance for completing this questionnaire. When you have finished, please mail it in the enclosed envelope.

INSTRUCTIONS: Please rate the services you received from our practice. Select the response that best describes your experience. If a question does not apply to you, please skip to the next question. Space is provided for you to comment on good or bad things that may have happened to you.

Please use black or blue ink to fill in the circle completely. Example: ●

ACCESS	Very Poor 1	Poor 2	Fair 3	Good 4	Very Good 5
1. Ease of arranging your video or telephone visit	O	O	O	O	O
2. Ease of contacting us (e.g. email, phone, web portal)	O	O	O	O	O

CARE PROVIDER	Very Poor 1	Poor 2	Fair 3	Good 4	Very Good 5
**DURING YOUR VIDEO OR TELEPHONE VISIT, YOUR CARE WAS PROVIDED PRIMARILY BY A DOCTOR, PHYSICIAN ASSISTANT (PA), NURSE PRACTITIONER (NP), OR MIDWIFE. PLEASE ANSWER THE FOLLOWING QUESTIONS WITH THAT HEALTH CARE PROVIDER IN MIND.**					
1. Concern the care provider showed for your questions or worries	O	O	O	O	O
2. Explanations the care provider gave you about your problem or condition	O	O	O	O	O
3. Care provider's efforts to include you in decisions about your care	O	O	O	O	O
4. Care provider's discussion of any proposed treatment (options, risks, benefits, etc.)	O	O	O	O	O

Supporting Information

5. Likelihood of your recommending this care provider to others	O	O	O	O	O

Comments (describe good or bad experience): ____

____

TELEMEDICINE TECHNOLOGY	Very Poor **1**	Poor **2**	Fair **3**	Good **4**	Very Good **5**
1. Ease of talking with the care provider over the video or audio connection	O	O	O	O	O
2. How well the audio connection worked during your visit	O	O	O	O	O
3. How well the video connection worked during your visit **(please only answer if your visit was by video)**	O	O	O	O	O

OVERALL ASSESSMENT	Very Poor **1**	Poor **2**	Fair **3**	Good **4**	Very Good **5**
1. How well the staff (including the care provider) worked together to care for you	O	O	O	O	O
2. Likelihood of your recommending our practice to others	O	O	O	O	O

ADDITIONAL COMMENTS

1. Please tell us about anything that impressed you about your experience.

   ____

   ____

   ____

2. Please tell us about anything that disappointed you about your experience.

   ____

   ____

   ____

Supporting Information

Supplemental table 4: How the frequency of top box score of 5 varied by month for telenephrology survey responses

	Telemedicine Survey Questions									Number of visits	
	Ease of talking with the care provider over the video or audio connection			How well the audio connection worked during your visit, whether by phone or video			If you had a video visit, how well the video connection worked			Phone n = 878	Video n = 353
**Month**	**Count of Top Box =5**	**Total**	**%**	**Count of Top Box =5**	**Total**	**%**	**Count to Top Box =5**	**Total**	**%**		
March	9	12	75%	9	12	75%				133	2
April	25	33	76%	26	33	79%	7	9	78%	363	63
May	13	15	87%	10	15	67%	4	7	57%	119	76
June	20	24	83%	20	24	83%	6	11	55%	153	102
July	14	15	93%	14	15	93%	12	13	92%	110	110

Supplemental Figure 1: How the frequency of top box score of 5 varied by month for telenephrology survey responses

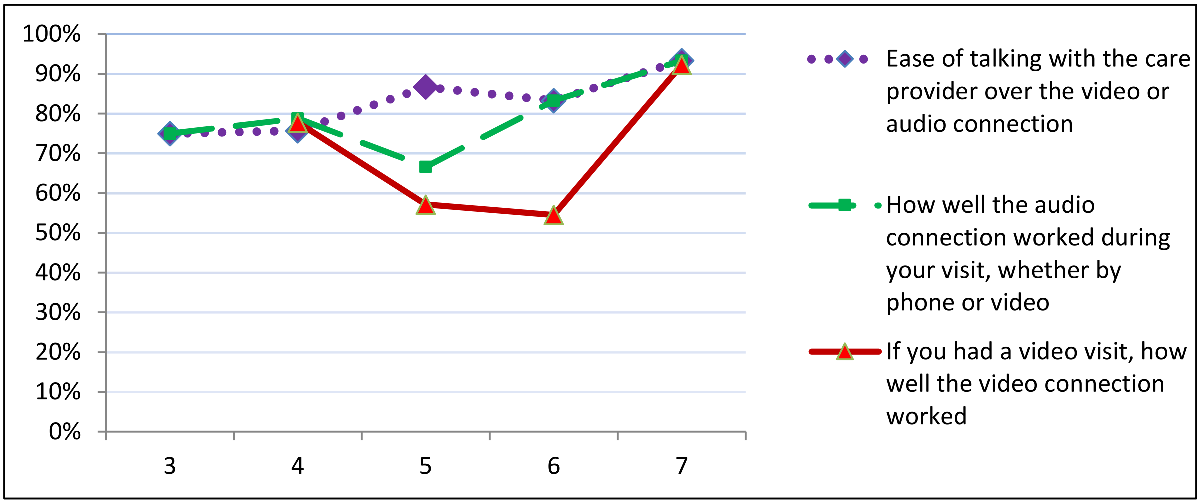

Supporting Information

Supplemental Table 5: **Press Ganey Survey - Medical Practice Survey Response Rate for October 2019 – September 2020, Nephrology Division, Mayo Clinic, Rochester MN**

Number of Providers	48
Number of surveys mailed	4663
Number of surveys undelivered	27
Number of surveys returned	1399
Average survey response rate	30.2%

Supplemental Figure 2: Survey respondents distribution by visit type and diagnoses groups (Face-to-face and telenephrology)

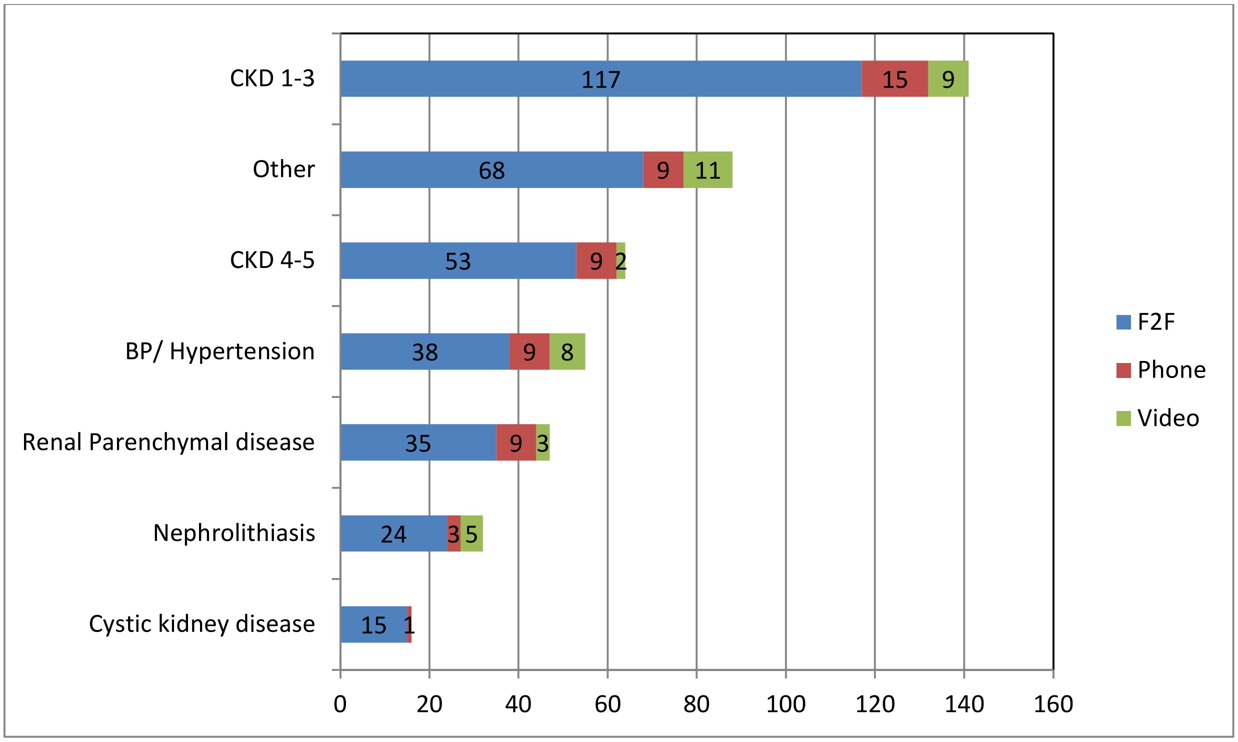

Supporting Information

Supplemental Table 6: Survey Respondents by primary diagnosis group

Primary Diagnosis Group	F2F	Phone	Video	Grand Total
Cystic kidney disease	15	1		16
Nephrolithiasis	24	3	5	32
Renal Parenchymal disease	35	9	3	47
BP/ Hypertension	38	9	8	55
CKD 4-5	53	9	2	64
Other	68	9	11	88
CKD 1-3	117	15	9	141
**Grand Total**	**350**	**55**	**38**	**443**
