# Supplementary material for: Telehealth versus face-to-face visits: A comprehensive outpatient perspective-based cohort study of patients with kidney disease
Source: PLoS One. 2022 Mar 11;17(3):e0265073. doi: 10.1371/journal.pone.0265073 (PMC8916620; doi:10.1371/journal.pone.0265073)
Supplement: Attachment — Submitted filename: Response to Reviewers.docx [file pone.0265073.s002.docx]

**Response to Reviewers**

Please see below for responses to the reviewers’ comments/ questions

Comments to the Author

**1. Is the manuscript technically sound, and do the data support the conclusions?**

The manuscript must describe a technically sound piece of scientific research with data that supports the conclusions. Experiments must have been conducted rigorously, with appropriate controls, replication, and sample sizes. The conclusions must be drawn appropriately based on the data presented.

Reviewer #1: Yes

Reviewer #2: Yes

________________________________________

**2. Has the statistical analysis been performed appropriately and rigorously?**

Reviewer #1: Yes

Reviewer #2: Yes

________________________________________

**3. Have the authors made all data underlying the findings in their manuscript fully available?**

The PLOS Data policy requires authors to make all data underlying the findings described in their manuscript fully available without restriction, with rare exception (please refer to the Data Availability Statement in the manuscript PDF file). The data should be provided as part of the manuscript or its supporting information, or deposited to a public repository. For example, in addition to summary statistics, the data points behind means, medians and variance measures should be available. If there are restrictions on publicly sharing data—e.g. participant privacy or use of data from a third party—those must be specified.

Reviewer #1: Yes

Reviewer #2: Yes

________________________________________

**4. Is the manuscript presented in an intelligible fashion and written in standard English?**

PLOS ONE does not copyedit accepted manuscripts, so the language in submitted articles must be clear, correct, and unambiguous. Any typographical or grammatical errors should be corrected at revision, so please note any specific errors here.

Reviewer #1: Yes

Reviewer #2: Yes

________________________________________

**5. Review Comments to the Author**

Please use the space provided to explain your answers to the questions above. You may also include additional comments for the author, including concerns about dual publication, research ethics, or publication ethics. (Please upload your review as an attachment if it exceeds 20,000 characters)

Reviewer #1: With the current pandemic the need for more telemedicine to serve the population has become more obvious. There are quite a few biases in the results but the authors do a good job of acknowledging them in their discussion which includes small survey size, mostly established patients.

Also given that the authors don't know if the survey pertained to specifically the nephrology visit would it not be prudent to change the title to NOT include nephrology?

Response: All the patients included in our study have kidney disease and visited with our Nephrology & Hypertension division at least once during the study period. These patients commonly have multiple comorbidities requiring care by a multidisciplinary team spanning various medical specialities. As such, we have changed the title of the paper to be “Telehealth versus Face-to-face visits: A Comprehensive Outpatient Patient Perspective-based Cohort Study of Patients with Kidney Disease”

Also most telehealth visit patients lived further away and are generally known to be more satisfied if they don't have to travel far to see their physician, I think that is worth a mention.

Response: This is true. We have now included it in the manuscript (fifth paragraph of discussion section).

And I like that they acknowledged that they couldn't measure patient outcomes which would be good to look at in the future and see if they correlated with the patient satisfaction scores

Response: Understanding patient outcomes, patient and provider perspectives on the different models of telehealth will be very important in figuring out how to improve care delivery to patients. It will be useful to study these factors not just within our institution but nationally and globally too.

Reviewer #2: This is a retrospective single center study using a standardized structured survey to assess patient satisfaction, comparing telenephrology versus face-to-face visits in outpatient nephrology and hypertension clinic. Patient satisfaction was equally high for the telenephrology and face-to-face visits. Telenephrology was more likely to be done for established patients and younger patients. The majority of patients receiving telenephrology were found to reside further from clinic.

These findings were not surprising, but this study confirmed what we experience. There were 52 providers in this study which provided good sampling of the variability among providers. The authors carefully designed the study to assess the survey. The authors also acknowledged the limitations of the study and discussed the future studies that are needed.

**Major points**

Who decided the modality for each visit, the provider or the patient?

Response: This was mostly based on patient preference. Providers encouraged initial consults to be face-to-face or video. Established patients could choose face-to-face, video or telephone, based on their preference and medical needs. Providers could triage the cases prior to the appointments being set and provide guidance on the appropriateness of the modality, after patients make their preferences known.

Why did the authors reduce a 5-point Likert scale to a binary scale (i.e., good (4) or very good (5) vs. 1-3)?

Response: Our institution uses Likert scale survey questionnaires to evaluate patient experiences. The results are then summarized using top-box score analyses that combines the top two responses to create a single number. The institution focuses on service, quality, and excellence and holds providers accountable to achieving top box score experience when it comes to patient satisfaction.

It may worthwhile to analyze the repeat visits.

Response: At the moment, we do not have enough data, given the study period, to effectively analyze repeat visits. The COVID-19 pandemic also continues to rage on, and this may affect patients’ perspectives on telehealth. However, as telehealth persists and patients continue to seek care at our institution, we will have the longitudinal data and we are excited to share our findings over time.

Could the authors further discuss the changes in telenephrology from pre-COVID to COVID times, in addition to the race differences mentioned? Are there any relevant studies that should be cited in this regard?

Response: Prior to the COVID-19 pandemic, CMS payment requirements limited telehealth reimbursement. As such, many patients and providers did not have access to telehealth. However, COVID-19 pandemic led to the presidential order that directed CMS to expand telehealth services under the 1135 waiver authority and Coronavirus Preparedness and Response Supplemental Appropriations Act. The opportunities to effectively study racial differences in the use of telehealth given access barriers, literacy/language issues along with acceptance is a goal this research team hopes to address in upcoming work.

**Minor**

Table 2 legend should be October 2019 - September, 2020.

Response: The study period was from March, 2020 – July, 2020

________________________________________

**6. PLOS authors have the option to publish the peer review history of their article (what does this mean?). If published, this will include your full peer review and any attached files.**

If you choose “no”, your identity will remain anonymous, but your review may still be made public.

Do you want your identity to be public for this peer review? For information about this choice, including consent withdrawal, please see our Privacy Policy.

Reviewer #1: No

Reviewer #2: No
